# Central wave reflection is associated with peripheral arterial resistance in addition to arterial stiffness in subjects without antihypertensive medication

**DOI:** 10.1186/s12872-016-0303-6

**Published:** 2016-06-07

**Authors:** Matias Wilenius, Antti J. Tikkakoski, Anna M. Tahvanainen, Antti Haring, Jenni Koskela, Heini Huhtala, Mika Kähönen, Tiit Kööbi, Jukka T. Mustonen, Ilkka H. Pörsti

**Affiliations:** School of Medicine, Department of Internal Medicine, University of Tampere, Tampere, FIN-33014 Finland; School of Medicine, Department of Pharmacological Sciences, University of Tampere, Tampere, Finland; School of Health Sciences, University of Tampere, Tampere, Finland; Department of Clinical Physiology, Tampere University Hospital, Tampere, Finland; Department of Internal Medicine, Tampere University Hospital, Tampere, Finland

**Keywords:** Augmentation index, Arterial stiffness, Central wave reflection, Systemic vascular resistance

## Abstract

**Background:**

Augmentation index, a marker of central wave reflection, is influenced by age, sex, height, blood pressure, heart rate, and arterial stiffness. However, the detailed haemodynamic determinants of augmentation index, and their relations, remain uncertain. We examined the association of augmentation index with vascular resistance and other haemodynamic and non-haemodynamic factors.

**Methods:**

Background information, laboratory values, and haemodynamics of 488 subjects (239 men, 249 women) without antihypertensive medication were obtained. Indices of central wave reflection, systemic vascular resistance, cardiac function, and pulse wave velocity were measured using continuous radial pulse wave analysis and whole-body impedance cardiography.

**Results:**

In a regression model including only haemodynamic variables, augmentation index in males and female subjects, respectively, was associated with systemic vascular resistance (β = 0.425, β = 0.336), pulse wave velocity (β = 0.409, β = 0.400) (*P* < 0.001 for all), stroke volume (β = 0.256, β = 0.278) (*P* = 0.001 for both) and heart rate (β = −0.150, β = −0.156) (*P* = 0.049 and *P* = 0.036). When age, height, weight, smoking habits, and laboratory values were included in the regression model, the most significant explanatory variables for augmentation index in males and females, respectively, were age (β = 0.577, β = 0.557) and systemic vascular resistance (β = 0.437, β = 0.295) (*P* < 0.001 for all). In the final regression model, pulse wave velocity was not a significant explanatory variable for augmentation index, probably due to the high correlation of this variable with age (Spearman’s correlation ≥0.617).

**Conclusion:**

Augmentation index is strongly associated with systemic vascular resistance in addition to arterial stiffness.

**Trial registration:**

ClinicalTrials.gov, NCT01742702.

**Electronic supplementary material:**

The online version of this article (doi:10.1186/s12872-016-0303-6) contains supplementary material, which is available to authorized users.

## Background

Reflected pressure waves from the peripheral circulation, originating from the arterial branches and resistance arteries, augment central blood pressure (BP) at the aortic root level [1]. Augmentation index (AIx), a widely used marker of central wave reflection, is defined as a ratio between augmentation pressure (contribution of the reflected pressure wave to systolic pressure) and pulse pressure. Ideally, AIx should be obtained at the central level, i.e. at the site of the carotid artery or ascending aorta [2]. AIx can be recorded non-invasively from the carotid artery with an applanation tonometry device [3], but is usually estimated from radial arterial tonometric signal using a generalized transfer function [4]. According to some studies, an increase in AIx has predictive value for future cardiovascular events and mortality [5–7]. However, this was not seen in the Framingham study, in which elevated pulse wave velocity (PWV), an acknowledged marker of arterial stiffness [2], was found to increase the risk of future cardiovascular morbidity [8].

Despite intensive research in the field of central wave reflection, the detailed determinants of AIx are still under debate. Naturally, there is a strong correlation between AIx and BP. AIx has shown to be higher among females when compared with males, and there is an inverse correlation between AIx and height [9, 10]. This inverse correlation is considered to result from earlier return of wave reflection in shorter subjects [10]. Age has been repeatedly shown to be a strong determinant of AIx [9, 11]. In addition, AIx has been reported to correlate inversely with heart rate (HR), so that it falls on average by 4 % for every 10 beats/min increase in HR [12].

Since central wave reflection is influenced by arterial compliance, AIx is often referred to as a marker of arterial stiffness [13–19]. However, according to expert consensus documents, AIx is an indirect surrogate measure of arterial stiffness that provides additional information concerning central wave reflections [2, 20].

Systemic vascular resistance (SVR) is of foremost importance in defining BP and haemodynamics, but this variable is hardly ever determined in clinical practice and seldom even in research settings [21]. Since the reflected pressure waves from the peripheral circulation significantly originate from the resistance arteries [1], it seems likely that higher SVR would lead to a more prominent wave reflection and higher AIx. Based on experiments utilizing the vasodilator nitroglycerin, a decrease in SVR has been considered to reduce AIx, although SVR was not actually measured in the experimental setting [22]. Actually, simultaneous evaluations of SVR and AIx have rarely been performed [21, 23, 24], and to our knowledge the association of AIx and SVR versus PWV has not been compared.

Taken together, there are still several unanswered questions regarding the determinants of AIx. In this study, we compared the role of SVR versus PWV as haemodynamic determinants of AIx by parallel measurements of these variables in 488 subjects.

## Methods

### Study subjects

Subjects to this study on haemodynamics were recruited from the University of Tampere, Tampere University Hospital, occupational health care providers, and by the use of two newspaper announcements (DYNAMIC-study, clinical trial registration NCT01742702). Also, people enrolling in a long-distance running program for beginners at Varala Sports Institute were informed about the study. The responding subjects from all of the above sources were recruited. Both normotensive and hypertensive individuals were allowed to participate. Before acceptance to the present study, lifestyle habits, medical history, and family history for cardiovascular disease were documented. Exercise habits were recorded as the number of self-reported ≥30 min exercise sessions per week that caused shortness of breath or sweating. A medical examination by a physician, and routine laboratory analyses of hypertension were performed [25]. By the time of the present study, 688 subjects had participated in the recordings. Subjects with antihypertensive medication, atrial fibrillation, or who had a history of diabetes, coronary artery disease, stroke, valvular heart disease or secondary hypertension were excluded from the present study. Altogether 488 subjects were included in this study.

### Laboratory analyses

Blood samples were obtained after approximately 12 h of fasting. A standard 12-lead electrocardiogram was recorded, and Cornell ECG voltage product was calculated. Plasma sodium, potassium, calcium, glucose, creatinine, cystatin-C, triglyceride, and total, high-density and low-density lipoprotein (HDL and LDL, respectively) cholesterol concentrations were determined by using Cobas Integra 700/800 or Cobas6000, module c501 (Roche Diagnostics, Basel, Switzerland). Blood cell count was analysed by ADVIA 120 or 2120 (Bayer Health Care, Tarrytown, NY, USA). Since creatinine values were within normal range, glomerular filtration rate was estimated using the Rule formula [26].

### Haemodynamic measurement protocol

Haemodynamics were recorded in a research laboratory by trained nurses. Study subjects were instructed to avoid caffeine containing products, smoking and heavy meals for at least 4 h prior to the investigation, whereas alcohol and heavy exercise were to be avoided for at least 24 h. Impedance cardiography electrodes were placed on body surface, tonometric sensor for pulse wave analysis on the radial pulsation to the left wrist, and an oscillometric brachial cuff for BP calibration to the right upper arm. The left arm (abducted to 90°) with the tonometric sensor rested on the level of the heart in an arm support [24]. The measurements were started once a visually assessed positional equilibrium was reached in the beat-to-beat measurement data. Haemodynamic data was recorded continuously for 5 min with the subjects in supine position.

### Pulse wave analysis

Radial BP and pulse waveform were determined from the radial pulsation by an automatic tonometric sensor (Colin BP-508 T, Colin Medical Instruments Corp., USA), calibrated approximately every 2.5 min by contralateral brachial systolic and diastolic BP measurements. Before the actual haemodynamic recordings, BP was measured manually 2 times by the use of an ordinary sphygmomanometer to verify that the automated BP readings are correct. Continuous aortic BP and wave reflections were derived from the radial tonometric signal with the SphygmoCor^R^ pulse wave monitoring system (SphygmoCor PWMx, AtCor Medical, Australia) using the previously validated generalized transfer function [4]. HR, aortic pulse pressure (aortic systolic pressure – aortic diastolic pressure), aortic augmentation pressure and AIx (aortic augmentation pressure/aortic pulse pressure × 100 %) were determined.

### Whole-body impedance cardiography

A whole-body impedance cardiography device (CircMon^R^, JR Medical Ltd, Tallinn, Estonia), which records the changes in body electrical impedance during cardiac cycles, was used to determine beat-to-beat HR, stroke volume, cardiac output, and aortic-popliteal PWV [27]. The mechanism of function, electrode placement, and processing of impedance cardiography data have been previously described [21, 27–29]. Briefly, the impedance cardiography method calculates PWV between the level of the aortic root and the popliteal artery by the use of the whole-body impedance signal and the signal measured from the popliteal artery region [27, 28]. The PWV results obtained using CircMon^R^ show good repeatability [29], and normal values for PWV in 799 individuals (age 25–76 years) have been previously published [30]. We have also shown that the determination of stroke volume using impedance cardiography versus 3-dimensional echocardiography show good correlation [29]. SVR was calculated from the tonometric radial BP signal and cardiac output measured by the CircMon^R^ device by subtracting average normal central venous pressure (4 mmHg) from mean arterial pressure and dividing it by cardiac output. Mean arterial pressure was calculated by using the formula: [(systolic BP)/3 + 2 × (diastolic BP)/3]. Cardiac output, stroke volume and SVR were indexed to body surface area (abbreviated as CI, SVI and SVRI, respectively).

### Whole-body impedance cardiography versus applanation tonometry in the measurement of PWV

The impedance cardiography method has been shown to agree well with Doppler ultrasound when assessing aortic-popliteal PWV [27]. However, the recording of aortic-popliteal PWV using impedance cardiography has not been previously compared with the gold standard method, tonometric carotid-femoral measurement of PWV. To examine the potential differences resulting from recordings in different large arterial segments (i.e. aortic-popliteal vs. carotid-femoral) on PWV, a series of recordings was performed to compare the measurements of PWV using the impedance cardiography versus the tonometric method in additional 80 volunteers. Cardiac arrhythmias, diagnosis of heart failure, carotid artery stenosis or valvular heart disease were used as exclusion criteria in this sub-study. The beat-to-beat recording of aortic-popliteal PWV using the CircMon^R^ impedance cardiography was carried out for one minute, and the carotid-femoral PWV using the SphygmoCor^R^ applanation tonometry was measured at the end of the same minute of recording. A previously validated equation was applied to adjust the PWV values obtained using impedance cardiography [27, 29]. The measurements were started once a visually assessed positional equilibrium was reached in the beat-to-beat measurement data.

### Statistical analyses

Mean values of the haemodynamic variables from the 3^rd^ to the 5^th^ minute of recording, when the signal was most stable, were used in the analyses. To study the determinants of AIx, linear regression analyses with backward elimination were performed separately for males and females because of the known difference in AIx between sexes [9]. Potential variables for multiple linear regression models were chosen after testing with univariate linear regression. All variables with *P* < 0.1 in either sex were included in the multivariate regression models. Although weight did not reach this criterion, it was included because of potential interrelation with several other variables. In the first regression model only haemodynamic variables (SVRI, PWV, HR, and SVI) were used as explanatory variables for AIx (Table 3). In the second regression model SVR, PWV, stroke volume, HR, age, height, weight, smoking (in pack years, i.e. number of packs containing 20 cigarettes smoked per day multiplied by years of smoking), plasma low-density lipoprotein (LDL) cholesterol, triglyceride, cystatin-C and fasting glucose concentrations were used as explanatory variables (Table 4). As the correlation between AIx and PWV was not linear, the common logarithm of PWV was used in the linear regression analyses. Coefficients (b) and standardized coefficients (beta) of regression, and Pearson’s correlations (r) were calculated. Spearman’s correlation was calculated for the association between AIx and PWV due to non-linearity.

In the PWV comparison study, each study subject’s beat-to-beat PWV using whole-body impedance cardiography during a one-minute period was recorded, and the average PWV was calculated. This mean PWV value was then compared with the PWV value measured using applanation tonometry at the end of the same one-minute period. Pearson’s correlation was calculated, and the two methods were compared using the statistical method of Bland and Altman [31].

Data were analysed using SPSS software version 17.0 (SPSS Inc., Chicago, Illinois, USA), presented as mean ± SD, and *p*-values <0.05 were considered significant.

## Results

### Subject characteristics and haemodynamics

The features of the study population (*n* = 488; 239 men and 249 women) are summarized in Table 1. The ten most common self-reported medical conditions were: dyslipidaemia (*n* = 101), asthma (*n* = 25), hypothyroidism (*n* = 17, all euthyroid), migraine (*n* = 15), gout (*n* = 14), osteoarthritis (*n* = 11), gastro-oesophageal reflux (*n* = 9), depression (*n* = 8), panic disorder (*n* = 5), and coeliac disease (*n* = 4). The majority of the subjects (*n* = 309/488) were completely without medications (for specific study subject medication details please see Additional file 1). Altogether 88 subjects were using female hormones (oestrogen, progestin or combination) for contraception or hormone replacement therapy (*n* = 37 with per oral tablets, *n* = 26 with levonorgestrel releasing intrauterine device). Although 101 subjects reported a history of dyslipidaemia, only 11 were using lipid-lowering medication.

**Table 1:** Basic characteristics and laboratory values in males and females

	Male	SD	Female	SD	*P*-value
	*n* = 239		*n* = 249		
Age (years)	46	11	45	12	0.498
Smoking (pack years)	3.4	9	1.3	6	0.002
Never (128 males, 157 females)	-		-		
Current (29 males, 26 females)	6.6	11.92	6.0	13.9	0.645
Previous (82 males, 65 females)	7.5	12.25	2.4	7.1	<0.001
Exercise habits (number of self-reported ≥30 min exercise sessions per week)	3.0	1.8	3.3	1.8	0.056
Height (cm)	180	6	166	6	<0.001
Weight (kg)	90	13	72	14	<0.001
Body mass index (kg/m^2^)	27.6	4.1	25.9	4.6	<0.001
Office systolic blood pressure (mmHg)	146	22	134	20	<0.001
Office diastolic blood pressure (mmHg)	92	11	85	12	<0.001
Cornell ECG voltage product (ms*mm)	1694	620	1609	482	0.093
Fasting plasma glucose (mmol/l)	5.6	0.6	5.3	0.5	<0.001
LDL cholesterol (mmol/l)	3.2	1.0	2.7	0.9	<0.001
HDL cholesterol	1.36	0.34	1.78	0.42	<0.001
Triglycerides (mmol/l)	1.50	1.33	1.06	0.63	<0.001
Cystatin-C (mg/l)	0.85	0.15	0.77	0.13	<0.001
eGFR (ml/min/1.73 m^2)^	119	14	104	9	<0.001

Values presented as means and standard deviations (SD); *LDL* low density lipoprotein, *HDL* high density lipoprotein, *eGFR*, rule formula of estimated glomerular filtration rate [28]

Since AIx is lower in males than females [9], the statistics were performed and scatter plot images were depicted separately for sexes. Apart from age, exercise habits and Cornell ECG voltage product, there was a statistically significant difference between males and females in every variable (Table 1).

Both systolic and diastolic BP was higher in males than females (Table 2). AIx and augmentation pressure were higher in females (*p* < 0.001 for both), whereas PWV (*p* < 0.001) and SVRI (*p* = 0.001) were higher in males. Apart from CI, all haemodynamic variables showed significant differences between the sexes (Table 2).

**Table 2:** Haemodynamic variables measured in the laboratory in males and females

	Male	SD	Female	SD	*P*-value
	*n* = 239		*n* = 249		
Systolic blood pressure (mmHg)	138	18	127	19	<0.001
Diastolic blood pressure (mmHg)	80	13	74	13	<0.001
Heart rate (1/min)	62	9	64	9	0.012
Augmentation index (%)	18.5	11.3	26.6	11.4	<0.001
Augmentation pressure (mmHg)	8.4	5.9	11.9	6.8	<0.001
Pulse wave velocity (m/s)	9.03	2.15	8.03	1.69	<0.001
Systematic vascular resistance (dyn*s/cm^5^)	1289	283	1402	319	<0.001
Systematic vascular resistance index (dyn*s/cm^5^/m^2^)	2686	583	2507	602	0.001
Stroke volume (ml)	98.1	13.5	80.7	13.6	<0.001
Stroke volume index (ml/m^2^)	47.0	6.6	45.2	7.3	0.004
Cardiac index (1/min/min^2^)	2.92	0.50	2.89	0.50	0.543

Values presented as means and standard deviations (SD)

### Correlations between AIx and other variables

**Fig. 1:**
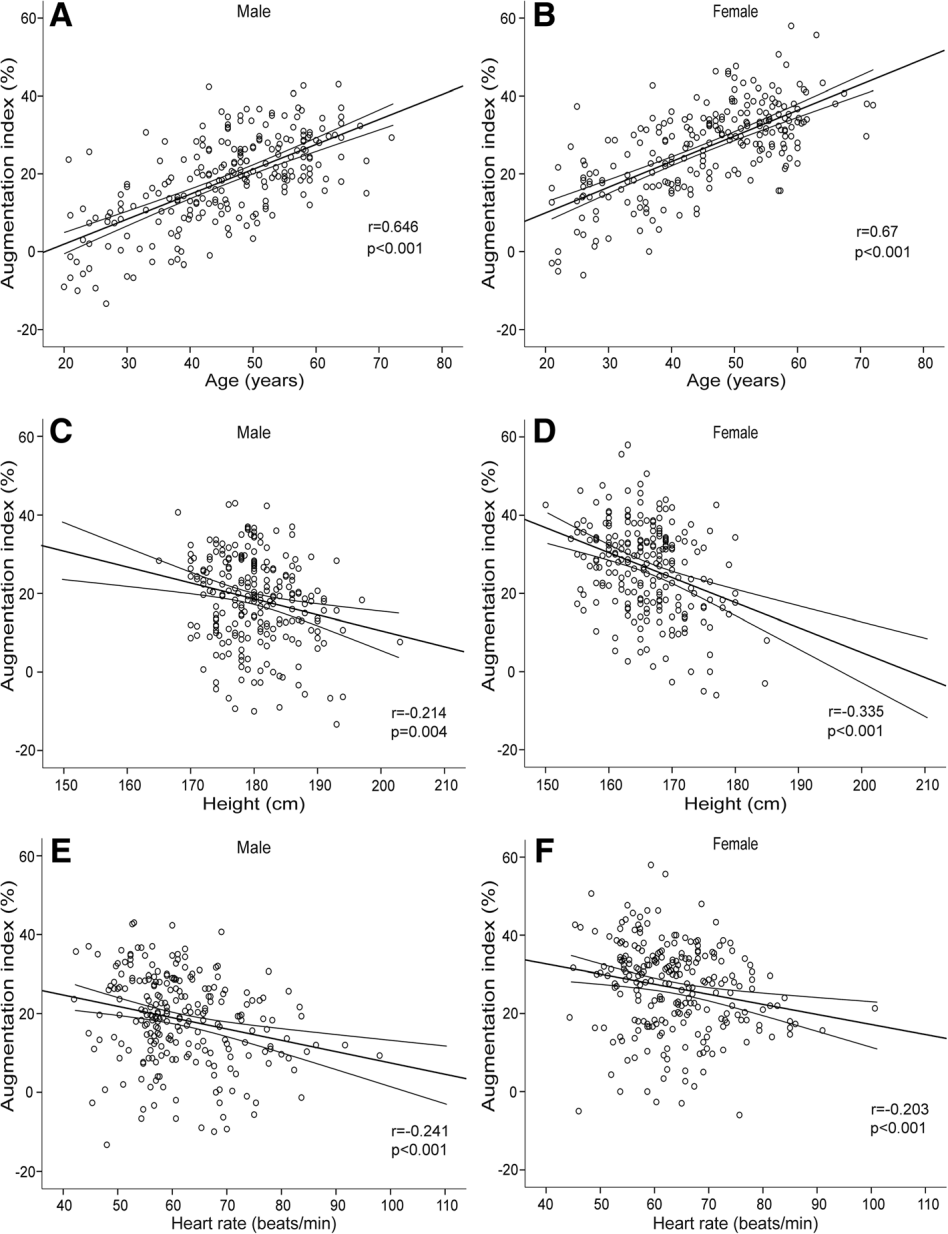
Associations between augmentation index and its known determinants. Scatter plots show associations between augmentation index and age (**a**, **b**), height (**c**, **d**), and heart rate (**e**, **f**) in male and female subjects, the lines depict mean and 95 % confidence intervals of mean

**Fig. 2:**
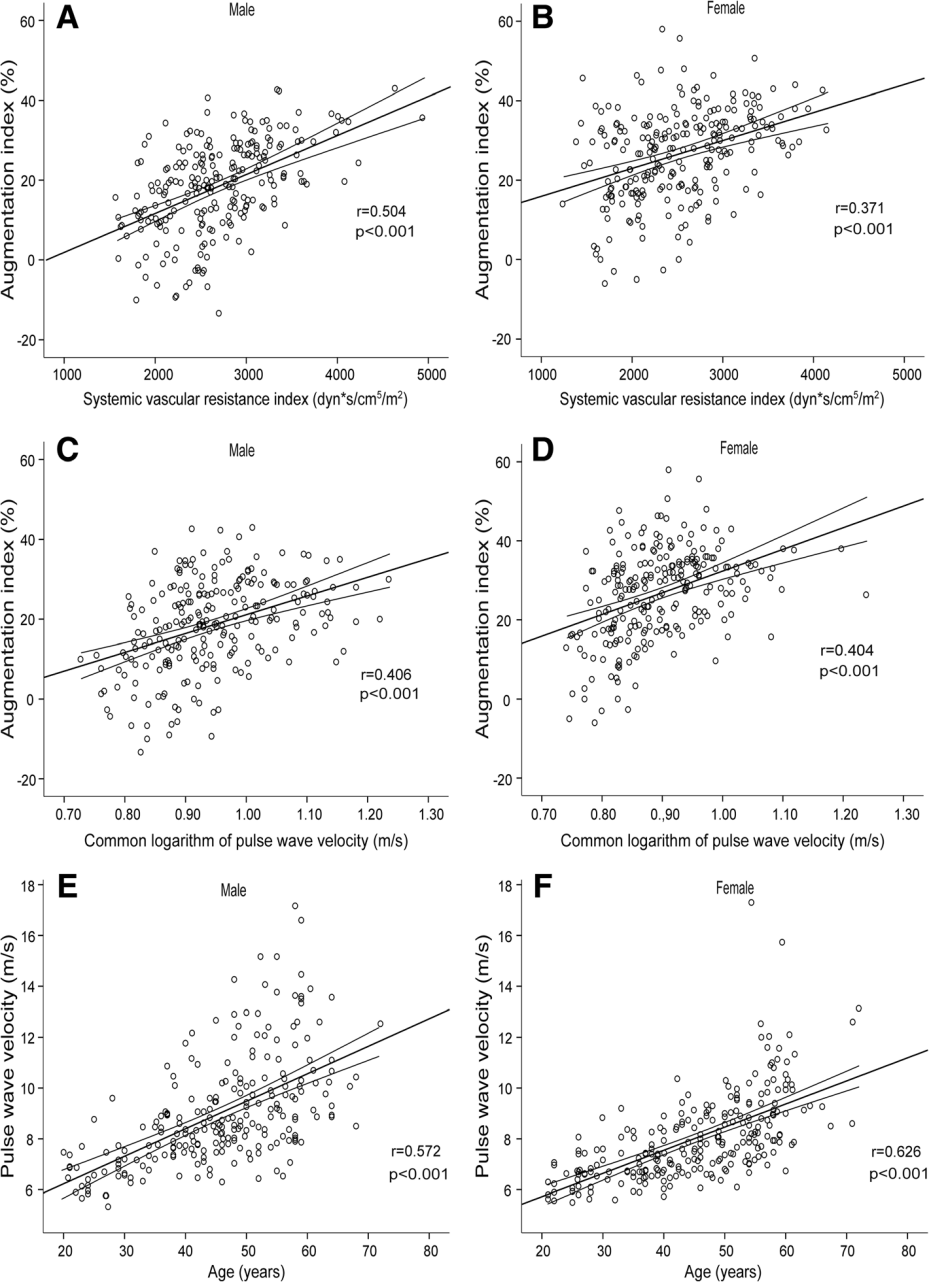
Associations between augmentation index and haemodynamic variables, and association between pulse wave velocity and age. Scatter plots show associations between augmentation index and systemic vascular resistance index (**a**, **b**), the common logarithm of pulse wave velocity (**c**, **d**), and association between pulse wave velocity and age (**e**, **f**) in male and female subjects, the lines depict mean and 95 % confidence intervals of mean

The association between AIx and age was very clear (Fig. 1a, b), and between AIx and height significant in both sexes, but more pronounced in females (Fig. 1c, d). The negative correlation between AIx and HR was modest but statistically significant (Fig. 1e, f). AIx showed a strong association with SVRI in males, while in females the association was somewhat less marked (Fig. 2a, b). The association between AIx and the common logarithm of PWV was corresponding in both sexes (Fig. 2c, d). As expected, age showed a strong correlation with PWV (Fig. 2e, f).

**Fig. 3:**
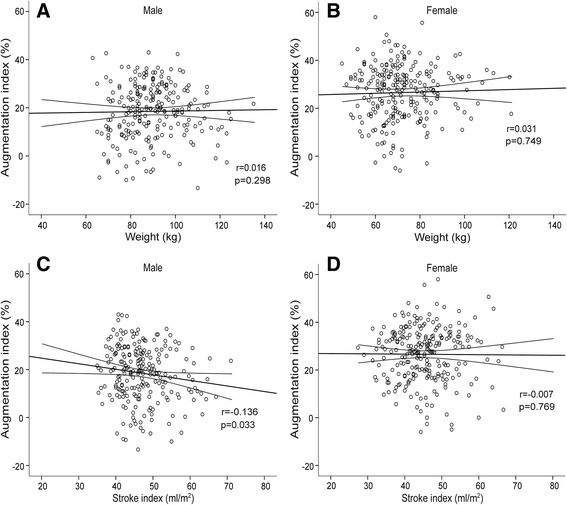
Associations between augmentation index and weight (**a**, **b**) and stroke volume index (**c**, **d**). Scatter plots in male and female subjects, the lines depict mean and 95 % confidence intervals of mean

AIx and weight showed no statistically significant correlation (Fig. 3a, b). SVI was not associated with AIx in females, whereas in males a weak inverse association was observed (*r* = -0.136, *p* = 0.033) (Fig. 3c, d). A Spearman’s correlation matrix for the haemodynamic variables and age for both sexes is presented in the supplemental material (Additional file 2).

### Regression analyses on factors associated with AIx

In the first regression analyses using only haemodynamic variables the results showed that, for both males and females, SVRI (*P* < 0.001 for both), PWV (*P* < 0.001 for both), SVI (*P* = 0.001 for both) and HR (*P* = 0.049 for males and *P* = 0.036 for females) were significant explanatory variables for AIx. The contributions of the beta values of the variables (SVRI, the common logarithm of PWV, HR and SVI) showed overlapping confidence intervals in this model. The overall R^2^ of the model was 0.381 in males and 0.317 in females (Table 3).

**Table 3:** Linear regression analysis with backward elimination: haemodynamic variables as explanatory variables for augmentation index

Augmentation index	b	beta	95 % Confidence interval of beta		*P* value
			Lower	Upper	
Male subjects, *R* ^*2*^ = 0.381, *p* < 0.001					
Systemic vascular resistance index	0.008	0.425	0.255	0.595	<0.001
L_g10_ (pulse wave velocity)	47.384	0.409	0.275	0.543	<0.001
Stroke volume index	0.434	0.256	0.104	0.407	0.001
Heart rate	-0.179	-0.150	-0.299	0.000	<0.049
Female subjects, *R* ^*2*^ = 0.317, *p* < 0.001					
L_g10_ (pulse wave velocity)	55.253	0.400	0.275	0.541	<0.001
Systematic vascular resistance index	0.006	0.336	0.157	0.515	<0.001
Stroke volume index	0.434	0.278	0.114	0.442	0.001
Heart rate	-0.199	-0.156	-0.303	-0.010	0.036

Variables used: Systematic vascular resistance index, the common logarithm of PWV, heart rate, stroke volume index. Lg10, the common logarithm

In the second regression model (Table 4), we used SVR and stroke volume instead of the indexed versions of these variables, as height and weight were also included in the model. SVR, the common logarithm of PWV, stroke volume, HR, age, height, weight, smoking in pack years, plasma LDL cholesterol, triglyceride, cystatin-C, and fasting glucose concentrations were used as explanatory variables. In male subjects, age, SVR, stroke volume and weight were significant explanatory variables for AIx (*R*^*2*^ = 0.580). In female subjects, age, SVR, height, stroke volume and smoking were the explanatory variables for AIx (*R*^*2*^ = 0.621). Of note, PWV was no more a significant explanatory variable for AIx in this model, probably due to the high correlation of this variable with age (Additional file 2).

**Table 4:** Regression analysis for determinants of augmentation index

Augmentation index	b	beta	95 % Confidence interval of beta		*P* value
			Lower	Upper	
Male subjects, R^2^ = 0.580, p < 0.001					
Age	0.563	0.577	0.460	0.671	<0.001
Systemic vascular resistance	0.018	0.437	0.354	0.631	<0.001
Stroke volume	0.160	0.185	0.068	0.314	0.003
Weight	-0.129	-0.131	-0.270	-0.032	0.013
Female subjects, *R* ^*2*^ = 0.621, *p* < 0.001					
Age	0557	0.557	0.474	0.670	<0.001
Systemic vascular resistance	0.011	0.295	0.180	0.400	<0.001
Height	-0.490	-0.255	-0.355	-0.161	<0.001
Stroke volume	0.154	0.177	0.067	0.302	0.002
Smoking	0.209	0.088	0.019	0.203	0.018

Linear regression analysis with backward elimination: haemodynamic variables, subject characteristics and laboratory values used as explanatory variables for augmentation index. Variables used: Systemic vascular resistance, the common logarithm of pulse wave velocity, stroke volume, heart rate, age, height, weight, smoking (in pack years); plasma LDL, triglyceride, cystatin-C and fasting glucose concentrations; LDL, low density lipoprotein

As several haemodynamic variables showed correlations with each other (Additional file 2), tests for collinearity were calculated in the regression models: variance inflation factors (VIF) were assessed for SVR, SVRI, and the common logarithm of PWV, the variables of interest. In all of the models, the VIF values ranged 1.5–2.8, which does not indicate a marked collinearity problem.

### Recording of PWV with impedance cardiography versus applanation tonometry

**Fig. 4:**
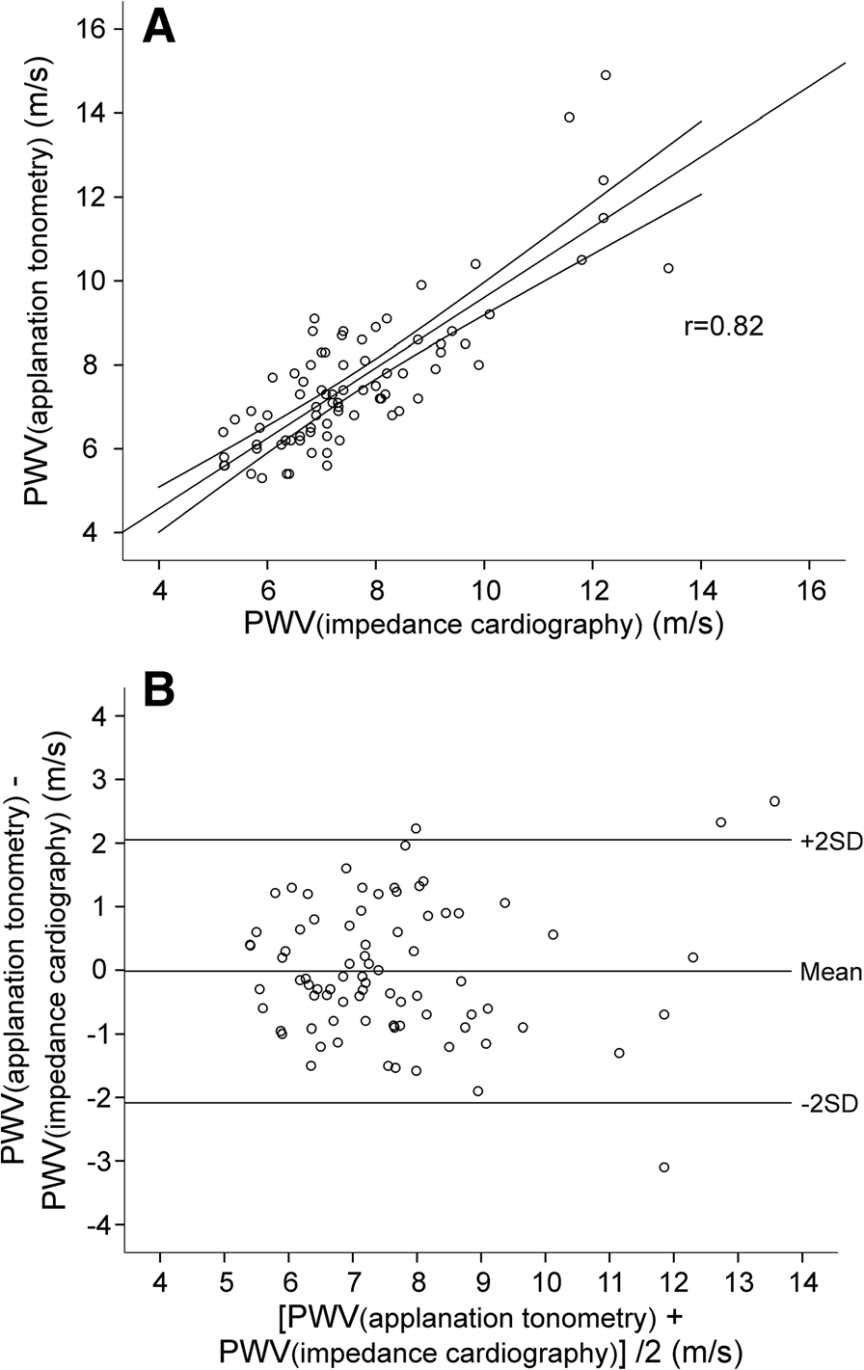
Comparison of two methods in the evaluation of large arterial stiffness. Correlation between pulse wave velocity measured using impedance cardiography and applanation tonometry, the lines depict mean and 95 % confidence intervals of mean (**a**); and differences between the two methods plotted against the average value of the methods with limits of agreement (±2SD) shown (**b**)

The basic characteristics of the additional 80 subjects participating in this separate sub-study are presented in the Additional file 3. The five most common self-reported medical conditions were hypertension (*n* = 16), asthma (*n* = 10), gastro-oesophageal reflux (*n* = 3), hypothyroidism (*n* = 3) and osteoarthritis (*n* = 2). Thirteen subjects were on BP-lowering medication. The mean carotid-femoral PWV measured using applanation tonometry was 7.63 m/s, whereas the mean aortic-popliteal PWV using impedance cardiography was 7.64 m/s. The bias (PWV(impedance cardiography) – PWV(applanation tonometry)) and precision (SD of differences) between these two methods were 0.02 and 1.06 m/s, respectively, and the correlation between these two methods was very good (Fig. 4a, b).

## Discussion

In the present study comprising a relatively large population (*n* = 488), AIx was significantly associated with peripheral vascular resistance in addition to PWV. As stiffening of the arteries results in earlier wave reflection and augmentation of central systolic pressure, AIx has often been labelled as a direct marker of arterial stiffness [13–19]. However, AIx is only an indirect measure of arterial stiffness that provides information concerning central wave reflections, whereas the determination of carotid-femoral PWV remains the gold standard for the evaluation of arterial stiffness [2, 20]. In order to determine the contribution of arterial stiffness to wave reflections, pulse wave analysis should be combined with the measurement of large arterial PWV [2].

Since the reflected pressure waves to a significant proportion originate from resistance arteries [1], SVR can be expected to affect wave reflection and correlate with the magnitude of AIx. The relative contributions of SVR versus large arterial stiffness as determinants of AIx have not been previously compared. In the present study, SVRI was a significant haemodynamic determinant of AIx, but due to overlapping confidence intervals the distinct order of the haemodynamic variables defining AIx cannot be stated (Table 3). In a regression model including haemodynamic and demographic data, SVR was the most significant haemodynamic determinant of AIx in males, whereas SVR and stroke volume were the significant haemodynamic determinants in females (Table 4). Like in previous studies [9–11], age and height were significantly related to AIx in both sexes. Although weight was a significant explanatory variable of AIx, this might rather be related subject height than adiposity, as the relationship was inverse, corresponding to the correlation between AIx and height in males (Fig. 1c). Of note, PWV was not a significant explanatory variable for AIx in the model that included several explanatory variables (Table 4). This can be attributed to the strong correlation between age and PWV (Fig. 2e, f). Recently, higher AIx was independently associated with greater media:lumen ratio in small subcutaneous arteries of 67 hypertensive subjects, while carotid-femoral PWV was not a significant explanatory variable for AIx in multiple regression analysis [32]. As remodelling of resistance arteries is an important determinant of vascular resistance [33], these results support the view of SVR as an important determinant of central wave reflection.

In addition to SVRI and PWV, SVI was a significant determinant of AIx in the regression analyses (Table 3). This might be due to the higher blood volume entering the aorta and the subsequent increase in the haemodynamic load, since the pattern of ventricular ejection is known to influence AIx [2]. Previous reports have also suggested that AIx is not only a measure of reflected pressure waves, but it also exhibits the properties of cardiac performance and ventricular-vascular coupling [23, 34, 35]. Yet, we also observed a weak inverse correlation between AIx and SVI in males (Fig. 3c). This could possibly be explained by the higher ejected blood volume increasing pressure at the first inflection point of the aortic pressure wave without affecting pressure at the second inflection point, the mechanism of which could result in a reduction of the augmented pressure.

Although the present results indicate that SVRI is significantly associated with AIx, they show no causalities, and a reverse connection could also be possible. Increased pulsatile pressure has been suggested to damage the peripheral vasculature causing vascular remodelling with a subsequent increase in arterial resistance [36]. We found a clear association between age and AIx (*r* = 0.646-0.67), and between age and PWV (*r* = 0.572-0.626), but the association between AIx and PWV was less marked (*r* = 0.361-0.376). Thus, age-related changes in AIx cannot be entirely explained by increased arterial stiffness, at least when stiffness is evaluated by measurement of PWV. The present haemodynamic measurements were continuous and data capture lasted for 3 min, and the statistical analyses of each variable in individual subjects were based on the mean of approximately 180–190 cardiac cycles. Many previous studies utilizing pulse wave analysis have obtained haemodynamic values with a pen-like tonometric sensor from 10 consecutive heart beats [14], which is a more operator dependent method. The aortic-popliteal impedance cardiography measurement of PWV has been previously validated against aortic-popliteal PWV measurements using Doppler ultrasound [27]. Our present recordings in 80 subjects showed no bias, and a very good correlation was observed between the tonometric (carotid-femoral) and the impedance cardiography (aortic-popliteal) measurements of PWV (Fig. 4), although these recordings are not focused on identical sections of the arterial tree. Finally, AIx is not an ideal measure of wave reflections [6, 7, 37], and additional studies that use wave separation to explore wave reflections are needed to corroborate the present findings in the future.

## Conclusions

We found that AIx was significantly determined by both SVRI and PWV. However, SVR was a more significant determinant in a model, where demographic, metabolic and haemodynamic factors were comprehensively considered. The present results add up to the data showing that AIx is a marker of central wave reflections, the magnitude of which is influenced by arterial stiffness.

## Abbreviations

AIx, augmentation index; BP, blood pressure; HDL, high-density lipoprotein; HR, heart rate; LDL, low-density lipoprotein; PWV, pulse wave velocity; SVI, stroke volume index; SVR, systemic vascular resistance; SVRI, systemic vascular resistance index

## Additional files

Additional file 1: Number of subjects with each type of medication. (DOCX 14.4 KB) Additional file 2: Spearman’s correlation matrix for the haemodynamic variables and age. (DOCX 14.5 KB) Additional file 3: Basic characteristics of 80 volunteers (45 females, 35 males) in the sub-study comparing pulse wave velocity measurements using whole-body impedance cardiography versus arterial tonometry. (DOCX 17.3 KB)
