# Supplementary material for: Central wave reflection is associated with peripheral arterial resistance in addition to arterial stiffness in subjects without antihypertensive medication
Source: BMC Cardiovasc Disord. 2016 Jun 7;16:131. doi: 10.1186/s12872-016-0303-6 (PMC4897906; doi:10.1186/s12872-016-0303-6)
Supplement: Additional file 1: — Number of subjects with each type of medication. (DOCX 14.4 KB) [file 12872_2016_303_MOESM1_ESM.docx]

**Additional file 1.** Number of subjects with each type of medication.

|  | n = 488 |
| --- | --- |
| Acetylsalicylic acid | 7 |
| Acyclovir | 2 |
| Alendronate | 1 |
| Antihistamine | 11 |
| Benzodiazepine | 2 |
| Carbamazepine | 1 |
| Carbimazole | 1 |
| Calcium carbonate | 7 |
| Female hormones |  |
| Oestrogen + progestin | 37 |
| Oestrogen | 14 |
| Progestin | 11 |
| Digoxin | 1 |
| Doxycycline (low dose) | 1 |
| Ezetimibe | 1 |
| Flecainide | 1 |
| Flupentixol | 1 |
| Folic acid supplementation | 1 |
| Glucosamine | 5 |
| Inhaled corticosteroids | 21 |
| Iron supplement | 2 |
| Levetiracetam | 1 |
| Lymecycline | 1 |
| Levonorgestrel via intrauterine device | 26 |
| Magnesium supplementation | 1 |
| Melatonin | 2 |
| Methenamine hippurate | 1 |
| Montelukast | 1 |
| Non-steroidal anti-inflammatory drugs | 4 |
| Oxcarbazepine | 1 |
| Potassium-phosphate | 1 |
| Pramipexole | 1 |
| Pregabalin | 1 |
| Proton pump inhibitor | 11 |
| Salbutamol (inhaled) | 2 |
| Salmeterol or formoterol (inhaled) | 7 |
| Sodium-phosphate | 1 |
| Statin | 11 |
| Sulfasalazine | 1 |
| Tacrolimus ointment | 1 |
| Tamoxifen | 1 |
| Tamsulosin | 2 |
| Thyroxin | 17 |
| Tibolone | 1 |
| Valproate | 1 |
| Varenicline | 1 |
| Vitamin B12 supplementation | 5 |
| Vitamin D supplementation | 15 |
| Warfarin | 1 |
