# Supplementary material for: Central wave reflection is associated with peripheral arterial resistance in addition to arterial stiffness in subjects without antihypertensive medication
Source: BMC Cardiovasc Disord. 2016 Jun 7;16:131. doi: 10.1186/s12872-016-0303-6 (PMC4897906; doi:10.1186/s12872-016-0303-6)
Supplement: Additional file 2: — Spearman’s correlation matrix for the haemodynamic variables and age. (DOCX 14.5 KB) [file 12872_2016_303_MOESM2_ESM.docx]

**Additional file 2.** Spearman’s correlation matrix for the haemodynamic variables and age.

|  | AIX | SVRI | HR | SVI | PWV | Age |
| --- | --- | --- | --- | --- | --- | --- |
| **Male subjects** |  |  |  |  |  |  |
| AIx | 1.000 | 0.509* | -0.246* | -0.139* | 0.424* | 0.621* |
| SVRI | 0.509* | 1.000 | -0.338* | -0.537* | 0.424* | 0.308* |
| HR | -0.246* | -0.338* | 1.000 | -0.247* | 0.306* | 0.083 |
| SVI | -0.139* | -0.537* | -0.247* | 1.000 | -0.505* | -0.141* |
| PWV | 0.424* | 0.424* | 0.306* | -0.505* | 1.000 | 0.617* |
| Age | 0.621* | 0.308* | 0.083 | -0.141* | 0.617* | 1.000 |
| **Female subjects** |  |  |  |  |  |  |
| AIx | 1.000 | 0.379* | -0.223* | 0.013 | 0.431* | 0.649* |
| SVRI | 0.379* | 1.000 | -0.210* | -0.609* | 0.434* | 0.312* |
| HR | -0.223* | -0.210* | 1.000 | -0.304* | 0.279* | -0.044 |
| SVI | 0.13 | -0.609* | -0.304* | 1.000 | -0.367* | -0.078 |
| PWV | 0.431* | 0.434* | 0.279* | -0.367* | 1.000 | 0.683* |
| Age | 0.649* | 0.312* | -0.044 | -0.078 | 0.683* | 1.000 |

AIx, augmentation index; SVRI, systemic vascular resistance index; HR, heart rate; SVI, stroke volume index; PWV, pulse wave velocity; *p<0.05.
