# Supplementary material for: Central wave reflection is associated with peripheral arterial resistance in addition to arterial stiffness in subjects without antihypertensive medication
Source: BMC Cardiovasc Disord. 2016 Jun 7;16:131. doi: 10.1186/s12872-016-0303-6 (PMC4897906; doi:10.1186/s12872-016-0303-6)
Supplement: Additional file 3: — Basic characteristics of 80 volunteers (45 females, 35 males) in the sub-study comparing pulse wave velocity measurements using whole-body impedance cardiography versus arterial tonometry. (DOCX 17.3 KB) [file 12872_2016_303_MOESM3_ESM.docx]

**Additional file 3.** Basic characteristics of 80 volunteers (45 females, 35 males) in the sub-study comparing pulse wave velocity measurements using whole-body impedance cardiography versus arterial tonometry.

|  | Mean ± SD | Min | Max |
| --- | --- | --- | --- |
| Age (years) | 43 ± 15 | 18 | 73 |
| Height (cm) | 172 ± 11 | 156 | 194 |
| Weight (kg) | 72 ± 14 | 49 | 122 |
| Body mass index (kg/m^2^) | 24 ± 3 | 18 | 33 |
| Office systolic blood pressure (mmHg) | 128 ± 16 | 99 | 183 |
| Office diastolic blood pressure (mmHg) | 76 ± 11 | 44 | 106 |
